# Evaluation of GCF Adiponectin and Resistin Expression Level as an Indicator in Periodontal Staging and Grading After Periodontal Therapy in Class III Obese Patients

**DOI:** 10.1155/ijod/6645532

**Published:** 2026-06-18

**Authors:** Bahaa Mohammed Badr, Ibrahim Hammad Ibrahim, Abdullah Ibrahim Ali, Shuaibu Abdullahi Hudu, Asmaa Rashad Ali, Asem Mohammed Kamel

**Affiliations:** ^1^ Department of Basic and Clinical Medical Science, Faculty of Dentistry, Zarqa University, Zarqa, Jordan, jadara.edu.jo; ^2^ Department of Medical Microbiology and Immunology, Faculty of Medicine, Al-Azhar University, Assiut, Egypt, azhar.edu.eg; ^3^ Department of Oral Medicine, Periodontology, Oral Diagnosis and Dental Radiology, Faculty of Dental Medicine, Al-Azhar University (Assiut Branch), Assiut, Egypt, azhar.edu.eg; ^4^ Center for Health Research, Northern Border University, Arar, 91431, Saudi Arabia, nbu.edu.sa; ^5^ Department of Microbiology, Faculty of Medicine, Northern Border University, Arar, 91431, Saudi Arabia, nbu.edu.sa; ^6^ Department of Medical Microbiology and Immunology, Faculty of Medicine for Girls (AFMG), Al-Azhar University, Cairo, Egypt, azhar.edu.eg

**Keywords:** adiponectin, obesity, periodontitis grading, resistin

## Abstract

**Background:**

The current trial aimed to assess the effects of phase I periodontal therapy on gingival crevicular fluid (GCF) adiponectin and resistin levels in obese individuals with varying stages and grades of periodontitis. The feasibility of using adiponectin and resistin in a periodontitis staging and grading classification system was being evaluated.

**Materials and Methods:**

This prospective clinical study was carried out on 60 class Ⅲ obese periodontitis patients who were divided equally into group І, first stage, grade B periodontitis; group Ⅱ, second stage, grade B periodontitis; group Ⅲ, third stage, grade B periodontitis; and group Ⅳ, fourth stage, grade C periodontitis. Body mass index (BMI), HbA1c, and periodontal indices were assessed, and GCF was collected and analyzed for adiponectin and resistin, before and 3 months after treatment.

**Results:**

A positive correlation was observed between BMI/HbA1c and periodontal disease stages. After periodontal therapy, adiponectin and resistin levels were obviously affected. In group Ⅲ resistin decreases from 25.68 ± 0.89 to 14.68 ± 1.54 ng/mL, adiponectin rose from 155.8 ± 39.7 to 435.8 ± 49.2 ng/mL. In group Ⅳ, resistin decreased from 53.2 ± 1.1 to 20.3 ± 2.03 ng/mL, and adiponectin increased from 104.41 ± 8.87 to 543.21 ± 36.81 ng/mL. There were no statistically significant correlations between clinical attachment level (CAL) and adiponectin/resistin. In grade B periodontitis, resistin decreased from 17.25 ± 6.1 to 13.5 ± 1.39 ng/mL after periodontal therapy. Adiponectin rose from 257.27 ± 84.26 to 355.61 ± 72.4 ng/mL. In grade C periodontitis, resistin expression was lower, from 53.2 ± 1.1 to 20.37 ± 2.03 ng/mL, whereas adiponectin was elevated from 104.41 ± 8.8 to 543.21 ± 36.81 ng/mL after 3 months of periodontal control treatment.

**Conclusion:**

Adiponectin and resistin were significantly affected after periodontal therapy, especially in stage 3 and stage 4 periodontitis. Adiponectin can be used as an intermediary for periodontitis grading.

## 1. Introduction

Periodontal disease taxonomy has been updated over recent years. The most widely adopted classification was announced in 1999 and remained in effect until 2017. Over time, worthwhile documentation has emerged from population trials, basic disciplinary investigations, and authentications from speculative research assessing environmental and systemic threat influencers. These attestations were presented during a 2017 workshop to propose a new classification for periodontitis [1]. By revising the classification of periodontitis, the workshop collaborated to develop a framework for its classification. It settled on a multifaceted staging and classification system that could be refined as new recommendations were published. Staging is principally based on the severity of disease at initial launch (“clinical epithelial attachment loss record”) and on the complexity of disease oversight (“bleeding on probing control”). Grading depends on appurtenant assessments of the nature of the disease, including a survey of the rate of bone loss, inquiries into modifiers of further disease progression, and encounters with therapy outcomes, which may negatively impact the systemic conditions of the individuals and vise versa [2, 3]. Various modifying factors were correlated with tooth deterioration and the progression of periodontitis. Obesity has been suggested to be a jeopardy element in periodontitis. Obesity is a complex condition with a wide range of causative factors, including genetic, biological, social, and behavioral factors, which intertwine to lead to a severe imbalance in energy metabolism ultimately. This discrepancy can lead to excessive fat accumulation and negative health consequences. Based on contemporary Health Canada Guidelines, a body mass index (BMI) (kg/m^2^) over 30 is categorized obese [4]. Class III obesity was strongly associated with an increased risk of mortality. The predominance of class III obesity is increasing rapidly because this drastic BMI is associated with severe health embarrassment, and the incidence of various diseases will increase substantially. Adipose tissue is a crucial endocrine organ that secretes adipocytokines or adipokines, which may link insulin resistance, obesity, and diabetes [5]. Among the known adipokines, adiponectin, leptin, resistin, IL‐6, TNF‐α, plasminogen activator inhibitor‐1, and monocyte chemoattractant protein‐1 are involved in the pathogenesis of insulin resistance in obesity. Adiponectin, a fat‐derived hormone, was discovered two decades ago. It served as a critical messenger in the crosstalk between the adipose tissue and other metabolism‐related organs [6]. Adiponectin provides a powerful shield against many pathological processes across various tissues by suppressing cell death, inhibiting inflammation, and promoting cell survival [7]. Reducing plasma adiponectin levels observed in patients with type 2 diabetes mellitus, metabolic syndrome, and coronary artery disease may be a crucial mandate in the evolution of insulin resistance. Adiponectin operates in a protective capacity against cardiovascular disorders, enhances insulin sensitivity, and has a beneficial effect on dietary lipid assimilation. Adiponectin expression decreased in obese individuals compared to nonobese, as well as in individuals pinpointed as insulin resistant; patients with type 2 diabetes mellitus and high blood pressure patients [8]. The known anti‐inflammatory properties of adiponectin and the long‐term inflammation in periodontitis suggest that adiponectin may have a protective function in periodontitis [9]. In 2001, resistin was portrayed during an exploration for genes that instigate adipocyte differentiation. Resistin and its family, resistin‐like signaling molecules (RELMs), are predominantly secreted by mononuclear cells, including neutrophils, macrophages, and monocytes. It has been recognized in the hypothalamus, adrenal glands, pancreas, gastrointestinal tract, myocytes, and spleen. Resistin is stimulated and released by various proinflammatory stimuli, such as TNF‐α, peptidoglycans, and bacterial endotoxins. Serum resistin levels are congruent with inflammatory and fibrinolytic mediators, specifically C‐reactive protein (CRP), TNF‐α, and IL‐6, in healthy individuals, patients with type 2 diabetes, coronary atherosclerosis, chronic kidney disease, and rheumatoid arthritis [10]. Resistin levels were elevated in individuals with obesity and prevalent disorders such as type 2 diabetes and rheumatoid arthritis. Furthermore, resistin was found to be elevated in gingival crevicular fluid (GCF) in patients with periodontitis, and therefore, it can be hypothesized that resistin increases periodontitis, thereby increasing the risk of periodontal disease or impaired periodontal healing [11]. The null presupposition for this trial sought behind that if there are any correlations amalgamate obesity markers (resistin/adiponectin) with the grading system of periodontitis. This scrutiny was designed to appraise the relationship between class Ⅲ obesity and GCF adiponectin and resistin levels in periodontitis patients beside estimating the consequence of nonsurgical periodontal therapy on their levels. Also, illustrating the ability to utilize the obesity markers in the classification of periodontitis staging and grading.

## 2. Materials and Methods

### 2.1. Ethical Considerations

The Helsinki Declaration ethical guidelines were followed in this trial, as its registration was conducted in the ClinicalTrials.gov Protocol Registration and Results System under ID NCT06604013. It was approved by the ethics committee of the Faculty of Dentistry, Al‐Azhar University, Assiut (approval number: AUAREC20230009‐4). Only adult contributors contributed voluntarily, and all of them provided written consent before volunteering for this trial.

### 2.2. Study Design

This prospective study was designed as a clinical comparative study, including patients with different stages of periodontitis. Participants were evaluated at baseline and re‐evaluated 3 months after nonsurgical periodontal therapy.

### 2.3. Sample Size Calculation and Power Analysis

The sample size calculation was done by 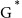
Power 3.1.9.2 (Universitat Kiel, Germany). We performed a pilot study (five cases in each group), and we found that the mean (±SD) of resistin levels was 13.3 ± 1.36 in mild periodontitis, 14.1 ± 2.9 in moderate periodontitis, 15.92 ± 2.5 in severe periodontitis, and 17.38 ± 4.57 in very severe periodontitis. The sample size was based on the following considerations: 0.567 effect size, 95% confidence limit, 90% power of the study, group ratio 1:1:1:1, and two cases were added to each group to overcome dropout. Therefore, we recruited 15 patients in each group.

### 2.4. Subjects

A total of 60 obese patients diagnosed with periodontitis (BMI 40–50 kg/m^2^, classified as class III obesity, was exclusively included to ensure a homogeneous study population and to evaluate the effects of severe obesity on periodontal and metabolic biomarkers without confounding from lower obesity classes) were recruited from those attended at the Outpatient Clinic, Oral Medicine and Periodontology Department, Faculty of Dental Medicine, Al‐Azhar University, Assiut, Egypt.

### 2.5. Obesity Assessment and Glycaemic Analysis

Participants were enrolled in these investigations according to the BMI scale, which is obtained by dividing body weight (kg) by the square of height (m) as a substitute for the total body fat measure. Obese patients were categorized according to the WHO (2021) as Type 1 (BMI ≥30 kg/m^2^ but <35), Type II (BMI ≥35 kg/m^2^ but <40), or Type III (BMI ≥40 kg/m^2^) [12]. Glycemic status was evaluated in all participants using glycated hemoglobin (HbA1c). Based on standard diagnostic criteria, participants were stratified as follows: HbA1c < 5.7% was considered normoglycemic, 5.7%–6.4% indicated prediabetes, and HbA1c ≥ 6.5% was considered diabetic. No participants were excluded based on HbA1c levels; instead, the glycemic status was recorded and analyzed in relation to periodontal disease severity and biomarker expression.

### 2.6. Grouping and Selection Benchmarks

Participants were grouped into four distinct sets of periodontitis according to clinical attachment level (CAL) quantification, as defined in the 2017 World Workshop on the Classification of Periodontal and Peri‐Implant Diseases and Conditions [13]. First group: 15 patients exhibited mild periodontitis (first stage, grade B), manifesting CAL ≥ 1–2 mm and ≥30% extensive periodontitis in dentition, bleeding on probing (BoP) (30%), in combination with a percentage of bone loss level divided by age of 0.25%–1% every year. Second group: 15 patients showing moderate periodontitis (second stage, grade B) manifesting CAL ≥ 3–4 mm and ≥ 30% stretched periodontitis in the mouth, BoP more than (30%) in correlation with the percentage of bone loss level divided by age was 0.25%–1% every year. Third group: 15 patients showing severe periodontitis (third stage, grade B) manifesting CAL ≥ 5 mm and ≥ 30% stretched periodontitis in the mouth, in corporation with BoP increased by more than (30%), the percentage of bone loss level divided by age was 0.25%–1% every year. Fourth group: 15 patients showing very severe periodontitis (fourth stage, grade C) manifesting CAL ≥ 5 mm and ≥ 30% spread periodontitis in the mouth, BoP was higher than (30%) in correlation with the percentage of bone loss level divided by age more than 1% every year.

Inclusion criteria included systemically obese adults diagnosed with periodontitis who had not received periodontal treatment within the previous 6 months.

Exclusion criteria of participants under any medicines for 3 months before investigations’ inception; conditions were accomplished: any periodontal prophylaxis in the last 6 months, exhaustive or compressed inflammatory changes aside from periodontitis, and finally any hormonal transposing conditions, such as lactating or being pregnant.

### 2.7. Periodontal Therapy

All periodontitis patients received nonsurgical full‐mouth periodontal therapy without the use of adjunct disinfectants. Supragingival scaling was performed with a Uds‐P LED Ultrasonic Scaler (Woodpecker Medical Instrument Co., Ltd., Guilin, Xiangshan District, China), and manual scaling was performed with a sickle scaler (Hu‐Friedy Manufacturing Co., LLC., Chicago, IL, USA). Subgingival scaling and root planing were performed with either universal or area‐specific Gracey curettes (Nordent Manufacturing Inc., Elk Grove Village, IL, USA).

### 2.8. Periodontal Assessment

Periodontal parameters were assessed at baseline and 3 months after periodontal therapy for all entities. Clinical parameters, including plaque scores (PIs), BoP percentage, CAL, and probing pocket depth (PPD), were reported. Pl was measured by O’Leary Pl as follows: bacterial deposits were stained with a disclosing solution to facilitate their detection. Calculation = the number of plaque‐containing surfaces/the total number of available surfaces. In the rating system, 0% indicates the absence of plaque, while 15%, 20%, and >40% indicate increasing plaque accumulation. BoP was performed at an expertise manual force of 0.25 N by a pressure of William’s graduated periodontal probe, recorded on distal, facial, mesial, and gingival surfaces. BoP was calculated as follows: number of bleeding surfaces/total number of tooth surfaces) multiplying by 100 and expressed in percentage (%). In the rating system, 0 indicates the absence of BoP, up to 10% (healthy), with 15% and 20% (gingivitis) indicating increased inflammation/infection, and > 20% indicating periodontitis. The PPD was measured from the gingival margin to the base of the pocket by William’s graduated periodontal probe. The attachment level was measured by subtracting the distance from the cementoenamel junction to the free gingival margin from the distance from the free gingival margin to the base of the pocket. Both were accurately measured using William’s graduated probe, and the difference between the two measurements yields the attachment level [14].

### 2.9. Gingival Crevicular Specimens’ Aggregation

The GCF level of adiponectin and resistin was assessed at baseline and 3 months after treatment. GCF samples were gathered from the site with the highest probing depth. Paragon areas were quarantined with cotton rolls and supragingival plaque was cleared out. Aridity measurements were done via an air syringe. Fluids were obtained by placing a paper point size #30, which was carefully inserted to the maximum depth of the periodontal pocket and held in position for 30 s. The assembled GCF was relocated in a flash into phosphate‐buffered saline (PBS) (137 mm NaCl, 10 mm Na_2_HPO_4_, and 2.7 mm KCl, pH 7.3) inside an Eppendorf tube, and then they were frozen till they were manipulated by commercially available enzyme‐linked immunosorbent assay (ELISA) (Shanghai Sunred Biological Technology Co., Ltd, Shanghai, China).

Samples of GCF contaminated with blood or saliva were excluded. The sampling area was isolated with cotton rolls and air‐dried to minimize contamination, and contaminated samples were recollected when possible.

### 2.10. Detection of Adiponectin and Resistin in GCF

Samples were assayed for resistin levels using two commercially available sandwich ELISA kits (Human Resistin & Human Adiponectin, ELISA Kit, Elabscience Bionovation Inc., Texas, USA) with a minimum detection limit of 78 pg/mL. The GCF samples were diluted with the dilution buffer in the kit, and the amounts of resistin and adiponectin determined according to the manufacturer’s instructions. Optical density (OD) is measured spectrophotometrically at 450 ± 2 nm. The OD value is proportional to the concentration of the human biomarker.

The primary outcome was the level of resistin in the GCF. Secondary outcomes included adiponectin levels and their association with periodontal parameters (PI, BoP, CAL, and PPD) and metabolic markers (BMI and HbA1c).

### 2.11. Statistical Analysis

The statistical analysis was performed using DATAtab: Online Statistics Calculator (DATAtab e.U. Graz, Austria). Shapiro–Wilks test and histograms were used to evaluate the normality of the distribution of data. Descriptive statistics were used for demonstrating mean and standard deviation values of demographic, clinical parameters, resistin, and adiponectin. *p*‐Values were adjusted for multiple comparisons using the Benjamini–Hochberg false discovery rate method. The Kolmogorov–Smirnov test was used to test the normality of the values. One‐way ANOVA was used to compare BMI with HbA1C, and a paired *t*‐test was used to compare them between groups. Bonferroni post hoc tests were used to compare periodontal parameters across groups. A Pearson correlation analysis was performed to assess the relationship between BMI and clinical parameters; a repeated—measures one—way ANOVA was used to assess adiponectin and resistin levels at different time points in the intergroup comparison. Paired *t*‐tests were used to compare adiponectin and resistin at different time points across all groups. Pearson correlation was used to compare adiponectin with resistin. A mixed‐model ANOVA was used to assess different periodontitis grades with respect to resistin and adiponectin. Statistical significance was set at *p*  < 0.05. Microsoft Excel was used to exemplify all charts and a heat map of correlations.

## 3. Results

### 3.1. Demographic Observations

**Figure 1:**
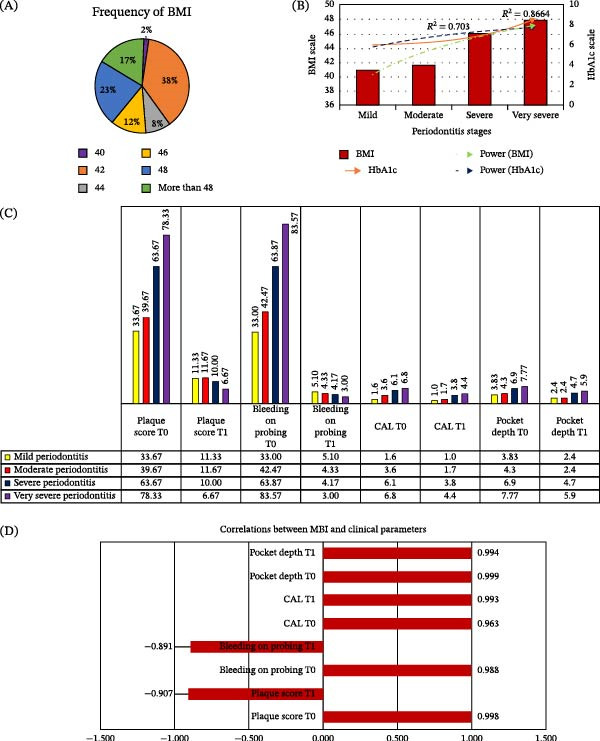
(A) Frequency of BMI in the study participants. (B) Correlations between BMI and HbA1c with different periodontitis stages. (C) Means of clinical parameters in all periodontitis stages at different intervals. (D) Correlations of clinical parameters with BMI at different intervals.

These investigations were done on 60 obese individuals aged from 33 to 67 years, with a mean age of 45.6 ± 8.5 years. Less than 30 years = 0.89%, less than 40 years = 27.14%, less than 50 years = 42.19%, less than 60 years = 24.71%, and less than 70 years = 5.07%. Both sexes (36 females and 24 males) were included in this trial (60% F and 40% M). Nonsmoking patients represented 70%, while 30% were smokers. BMI and HbA1c were estimated in all periodontitis patients. In mild periodontitis (first stage, grade B), BMI and HbA1c were 40.8 ± 1.14 and 6.04 ± 0.2, respectively. In moderate periodontitis (second stage, grade B), BMI and HbA1c showed no change, with values of 41.6 ± 0.62 and 6.18 ± 0.2, respectively. The apparent elevation of BMI and HbA1c was observed in severe periodontitis (third stage, grade B), with values of 46 ± 1.3 and 6.8 ± 0.12, respectively. The highest values of BMI and HbA1c were observed in very severe periodontitis (fourth stage, grade C), reaching 47.93 ± 0.87 and 8.62 ± 0.57, respectively. About 38% of participants had a BMI of 42–44, 23% had a BMI of 48, and 17% had a BMI greater than 48. Fewer proportions 12%, 8%, and 2% of patients had 46–48, 44−46, 40−42 BMI, respectively (Figure 1A).

### 3.2. BMI and HbA1c Correlation With Periodontitis Stage

There was a statistically significant difference between groups in BMI and HbA1c (*p*  < 0.001). In pairwise comparisons, there was a significant statistical difference between the third and fourth groups and the first group (*p*  < 0.001). In contrast, no significant difference was observed between the second group and the first group (*p* = 0.15 for BMI, *p* = 0.62 for HbA1c). Moreover, a statistically significant difference was found between the third and fourth groups and the second group (*p*  < 0.001). Finally, a statistically significant difference was found between the fourth group and the third group (*p*  < 0.001) (Table 1). A positive correlation was observed between BMI/HbA1c and the stage of periodontitis (Figure 1B).

**Table 1:** Comparing BMI and HbA1c in all entities and inter‐entities using one‐way ANOVA post hoc test.

One‐way ANOVA test					
	SS	df	MS	*F*	*p*
BMI	531.65	3	177.21	170.71	0.00 ^∗^
HbA1c	63.36	3	21.12	198.69	0.00 ^∗^

Groups juxtaposition			*t*	*p*	
A vs. B		BMI	2.15	0.21	
		HbA1c	1.18	0.99	
A vs. C		BMI	13.98	0.00 ^∗^	
		HbA1c	6.44	0.00 ^∗^	
A vs. D		BMI	19.17	0.00 ^∗^	
		HbA1c	21.67	0.00 ^∗^	
B vs. C		BMI	11.83	0.00 ^∗^	
		HbA1c	5.2	0.00 ^∗^	
B vs. D		BMI	17.02	0.00 ^∗^	
		HbA1c	20.49	0.00 ^∗^	
C vs. D		BMI	5.2	0.00 ^∗^	
		HbA1c	15.23	0.00 ^∗^	

*Note:* A—group І, B—group Ⅱ, C—group Ⅲ, D—group Ⅳ.

Abbreviations: ANOVA, analysis of variances; df, degrees of freedom; MS, mean square; SS, sum of squares.

^∗^ = ≤0.05 Significant.

### 3.3. Clinical Parameters Reflecting Periodontal Status

Under the influence of periodontal control by supra/subgingival scaling, all clinical periodontal parameters, including PIs, BoP percentage, CAL, and PPD, were improved after 3 months of evaluation. O’Leary plaque score was belittled by 66.35% from 33.67 ± 1.79 to 11.33 ± 1.14 in mild periodontitis (first stage, grade B), by 70.60% from 39.67 ± 1.24 to 11.67 ± 0.80 in moderate periodontitis (second stage, grade B), by 84.30% from 63.67 ± 1.86 to 10 ± 0.49 in severe periodontitis (third stage, grade B), and finally in the fourth group by 91.48% from 78.33 ± 1.93 to 6.67 ± 0.63. BoP percentages decreased successfully in all groups. In the first group, the change was from 33 ± 0.5 to 5.1 ± 0.51, in the second group, the change was from 42.47 ± 1.81 to 4.33 ± 0.4, in severe periodontitis (third stage, grade B) transposed to 4.17 ± 0.46 from 63.87 ± 1.64, and in the last group “very severe periodontitis (fourth stage, grade C)” BoP inverted from 83.57 ± 1.61 to minute worth 3.00 ± 0.46. When periodontal parameters were compared in all groups by using the ANOVA test, the assessments showed a statistically significant difference was found between all groups at the beginning of this trial and 3 months after periodontal therapy (Figure 1C, Table 2).

**Table 2:** Clinical parameters comparisons in all/in‐between groups using one‐way ANOVA post hoc Tukey test.

One‐way ANOVA for all groups																
			SS					MS			*F*			*p*		
PI T0			1956					6521.6			145.8			0.00 ^∗^		
PI T1			234.5					78.1			8.1			0.00 ^∗^		
BoP T0			23004					7668.2			232.8			0.00 ^∗^		
BoP T1			33.8					11.2			3.61			0.01 ^∗^		
CAL T0			254.4					84.8			263.3			0.00 ^∗^		
CAL T1			116					38.6			83			0.00 ^∗^		
PD T0			157.5					52.5			190.2			0.00 ^∗^		
PD T1			131					43.6			132.7			0.00 ^∗^		

Bonferroni post hoc tests for intergroups comparison																
	A vs. B			A vs. C		A vs. D			B vs. C			B vs. D			C vs. D	
	*t*	*p*		*t*	*p*	*t*	*p*		*t*	*p*		*t*	*p*		*t*	*p*
PI T0	2.46	0.1		12.2	0.00 ^∗^	18.3	0.00 ^∗^		9.83	0.00 ^∗^		15.8	0.00 ^∗^		6.1	0.00 ^∗^
PI T1	0.29	0.9		1.18	0.9	4.12	0.00 ^∗^		1.47	0.88		4.4	0.00 ^∗^		2.9	0.02 ^∗^
BoP T0	4.52	0.00 ^∗^		14.73	0.00 ^∗^	24.13	0.00 ^∗^		10.21	0.00 ^∗^		19.6	0.00 ^∗^		9.4	0.00 ^∗^
BoP T1	1.19	0.9		1.45	0.92	3.26	0.01 ^∗^		0.26	0.9		2.07	0.26		1.8	0.45
CAL T0	9.65	0.00 ^∗^		21.5	0.00 ^∗^	25.1	0.00 ^∗^		11.9	0.00 ^∗^		15.4	0.00 ^∗^		3.5	0.00 ^∗^
CAL T1	11	0.00 ^∗^		11.60	0.00 ^∗^	10.98	0.00 ^∗^		8.79	0.00 ^∗^		9.32	0.00 ^∗^		2.7	0.09
PD T0	2.49	0.09		16.35	0.00 ^∗^	20.9	0.00 ^∗^		13.8	0.00 ^∗^		18.4	0.00 ^∗^		4.6	0.00 ^∗^
PD T1	0.01	0.9		11.05	0.00 ^∗^	16.8	0.00 ^∗^		11.5	0.00 ^∗^		16.8	0.00 ^∗^		5.7	0.00 ^∗^

*Note:* T0—baseline, T1—after 3 Months, A—group І, B—group Ⅱ, C—group Ⅲ, D—group Ⅳ.

Abbrevitions: ANOVA, analysis of variances; df, degrees of freedom; MS, mean square; SS, sum of squares.

^∗^ = ≤0.05 Significant.

### 3.4. Correlation Between BMI and Clinical Parameters

There was a significant positive correlation between BMI and irreversible periodontal indicators (CAL and PD) at initial investigations and after 3 months of periodontal therapy. A significant positive correlation between MBI and reversible periodontal indicators (plaque score, BoP) was reported at initial investigations, whereas a negative correlation was observed 3 months after periodontal therapy (Figure 1D).

### 3.5. Adiponectin and Resistin Levels in Different Entities

In the mild periodontitis group, resistin decreased slightly from 12.77 ± 1.04 ng/mL before periodontal therapy to 12.6 ± 0.85 ng/mL after it. In contrast, adiponectin decreased from 341.1 ± 29.06 ng/mL before periodontal therapy to a minutely lessened 337.36 ± 36.5 ng/mL after periodontal therapy. In the moderate periodontitis group, resistin decreased slightly from 13.31 ± 0.9 ng/mL to 13.22 ± 0.75 ng/mL after periodontal therapy, and adiponectin increased from 274.83 ± 31.83 ng/mL to 293.6 ± 36.61 ng/mL after periodontal therapy. In severe periodontitis patients, resistin decreased from 25.68 ± 0.89 ng/mL at initial assessment to 14.68 ± 1.54 ng/mL after periodontal therapy, whereas adiponectin rose from 155.87 ± 39.77 to 435.87 ± 49.22 ng/mL after periodontal control. In very severe periodontitis patients, dramatic changes in resistin were observed, with a marked decrease from 53.2 ± 1.16 to 20.37 ± 2.03 ng/mL after periodontal therapy. Also, substantial increases in adiponectin were observed, with adiponectin increasing from 104.41 ± 8.87 to 543.21 ± 36.81 ng/mL with periodontal therapy hegemony (Table 3).

**Table 3:** Mean, standard deviation, and repeated‐measures one‐way ANOVA for resistin and adiponectin.

Stage	Mild			Moderate		Severe		Very severe	
Time point	T0	T 1		T0	T 1	T0	T1	T0	T1
Resistin (mean ± std.)	12.77 ± 1.04	12.6 ± 0.85		13.31 ± 0.9	13.22 ± 0.75	25.68 ± 0.89	14.68 ± 1.54	53.2 ± 1.16	20.37 ± 2.03
Adiponectin (mean ± std.)	341.1 ± 29.6	337.36 ± 36.5		274.8 ± 31.8	293.6 ± 36.61	155.87 ± 39.77	435.87 ± 49.22	104.41 ± 8.87	543.21 ± 36.81

Repeated measures one‐way ANOVA for resistin									
SS		df		MS		*F*		*p*	
16138.72		3		5379.57		4364.72		0.00 ^∗^	

Bonferroni post hoc tests for resistin									
Groups juxtaposition		Baseline				3 months			
		*t*	*p*			*t*		*p*	
Mild vs. moderate		1.371	0.9			2.119		0.314	
Mild vs. severe		29.713	0.00 ^∗^			4.465		0.00 ^∗^	
Mild vs. very severe		91.791	0.00 ^∗^			15.365		0.00 ^∗^	
Moderate vs. severe		38.943	0.00 ^∗^			3.454		0.023	
Moderate vs. very severe		90.412	0.00 ^∗^			12.686		0.00 ^∗^	
Severe vs. very severe		71.665	0.00 ^∗^			7.753		0.00 ^∗^	

Repeated measures one‐way ANOVA for adiponectin									
SS		df		MS		*F*		*p*	
527124.85		3		175708.28		271.82		0.00 ^∗^	

Bonferroni post hoc tests for adiponectin									
Groups juxtaposition		Baseline				3 months			
		*t*	*p*			*t*		*p*	
Mild vs. moderate		8.849	0.00 ^∗^			4.311		0.00 ^∗^	
Mild vs. severe		17.633	0.00 ^∗^			5.837		0.00 ^∗^	
Mild vs. very severe		30.345	0.00 ^∗^			16.432		0.00 ^∗^	
Moderate vs. severe		10.935	0.00 ^∗^			8.694		0.00 ^∗^	
Moderate vs. very severe		18.928	0.00 ^∗^			18.92		0.00 ^∗^	
Severe vs. very severe		5.412	0.00 ^∗^			16.135		0.00 ^∗^	

*Note:* T0—baseline, T1—after 3 Months, A—group І, B—group Ⅱ, C—group Ⅲ, D—group Ⅳ.

Abbreviations: ANOVA, analysis of variances; df, degrees of freedom; MS, mean square; SS, sum of squares.

^∗^ = ≤0.05 Significant.

### 3.6. Effects of Periodontal Therapy on the Standards of Adiponectin and Resistin

When investigating resistin in the mild group at two different time points, the *t*‐value of 0.71 indicated a small difference between the intervals. The *p*‐value (0.4) suggested no statistically significant difference between T0 and T1. Pearson correlation indicated a statistically significant, highly positive relationship between T0 and T1, showing that as T0 is elevated, T1 tends to remain high after periodontal therapy. In the moderate periodontitis group, the *t*‐test (*t* = 0.91) also indicated a small difference between the two intervals. The *p*‐value (0.3) showed no statistically significant difference between T0 and T1. Pearson correlation demonstrated a statistically significant, very high positive relationship, indicating that both resistin T0 and T1 tend to increase similarly. In the severe group, the *t*‐test (*t* = 21.5) showed a marked difference between T0 and T1, with a statistically significant *p*‐value (0.001). Pearson correlation was not statistically significant and showed a low negative relationship between T0 and T1, indicating that resistin increased at baseline and tended to decrease after periodontal therapy. In the very severe periodontitis group, the *t*‐value (58.01) indicated the greatest difference between the two time points, with a statistically significant *p*‐value (<0.05). Pearson correlation was not statistically significant, showing no meaningful positive association between T0 and T1. Resistin levels remained high before and after treatment, suggesting a more stable pattern in this group. Regarding adiponectin in the mild group, the *t*‐value (0.61) indicated a small difference between baseline and 3 months. The *p*‐value showed no statistically significant difference. Pearson correlation revealed a statistically significant, very high positive relationship, suggesting a consistent increase after therapy. In the moderate group, the *t*‐value (−5.98) indicated a greater difference between time points, with a statistically significant *p*‐value (<0.05). Pearson correlation showed a statistically significant, very high positive association. Adiponectin increased from baseline to 3 months, possibly reflecting anti‐inflammatory effects. In the severe group, the *t*‐value (−19.34) indicated a marked difference between intervals, with a statistically significant *p*‐value (<0.001). Correlation analysis showed no statistically significant relationship between baseline and 3 months. Adiponectin levels were lower at baseline and increased after periodontal therapy. In the very severe group, the *t*‐statistic (−50.55) indicated the greatest difference between baseline and 3 months, with a statistically significant *p*‐value (<0.05). Correlation analysis did not show a statistically significant association. Adiponectin increased after periodontal therapy, likely reflecting the improvement in inflammatory status and periodontal healing (Table 3).

### 3.7. Correlation of Adiponectin With Resistin in Different Intervals

In the mild group at baseline, there was a moderate, positive interconnection which was not statistically significant (*r* = 0.42, *p* = 0.1). At a 3‐month interval, there was a low, positive interconnection. A nonstatistically significant correlation was reported (*r* = 0.23, *p* = 0.4). In the moderate group at baseline, there was a high, statistically significant positive correlation (*r* = 0.63, *p* = 0.013). At a 3‐month interval, a moderate, positive correlation with no statistical significance (*r* = 0.49, *p* = 0.06). In the severe group at baseline, a low, negative interdependence was observed, with no statistically significant correlation (*r* = −0.21, *p* = 0.4). After a 3‐month interval from periodontal therapy, a high negative interdependence emerged, with a statistically significant correlation (*r* = −0.64, *p* = 0.01). In the very severe group at baseline, Pearson’s correlation showed a negligible, negative association that was not statistically significant (*r* = −0.04, *p* = 0.8). At a 3‐month interval, a low, positive correlation was detected, though not statistically significant (*r* = 0.27, *p* = 0.3) (Table 4).

**Table 4:** Comparison of levels of resistin and adiponectin before and after periodontal therapy by *t*‐test for paired samples and their correlations.

Resistin					
Baseline vs. 3 months	*t*	*p*	Cohen’s *d*	Correlation	
				*r*	*p*
Mild	0.71	0.48	0.18	0.54	0.038
Moderate	0.91	0.38	0.23	0.9	0.00 ^∗^
Severe	21.5	0.00 ^∗^	5.57	0.27	0.328
Very severe	58.01	0.00 ^∗^	14.98	0.14	0.628

Adiponectin					
Baseline vs. 3 months	*t*	*p*	Cohen’s *d*	Correlation	
				*r*	*p*
Mild	0.61	0.549	0.16	0.76	0.00 ^∗^
Moderate	−5.98	0.00 ^∗^	1.55	0.95	0.00 ^∗^
Severe	−19.34	0.00 ^∗^	4.99	0.22	0.431
Very severe	−50.55	0.00 ^∗^	13.05	0.46	0.081

Resistin vs. adiponectin Pearson correlation					
Baseline vs. 3 months		*r*		*p*	
Mild T0		0.42		0.11	
Mild T1		0.23		0.40	
Moderate T0		0.63		0.01 ^∗^	
Moderate T1		0.49		0.06	
Severe T0		−0.21		0.45	
Severe T1		−0.64		0.01 ^∗^	
Very severe T0		−0.04		0.87	
Very severe T1		0.27		0.33	

*Note:* T0—baseline, T1—after 3 Months, A—group І, B—group Ⅱ, C—group Ⅲ, D—group Ⅳ, Cohen’s *d*, standardized mean difference.

Abbreviations: ANOVA, analysis of variances; df, degrees of freedom; MS, mean square; SS, sum of squares.

^∗^ = ≤0.05 Significant.

### 3.8. Correlation Between CAL, Adiponectin, and Resistin

**Figure 2:**
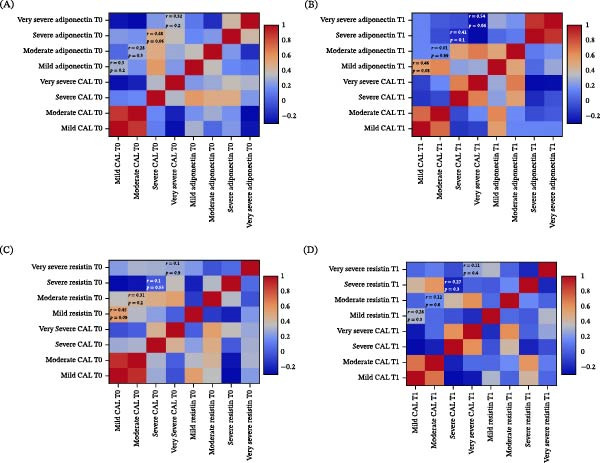
(A) Heatmap showing correlation of CAL with adiponectin at baseline. (B) Heatmap showing correlation of CAL with adiponectin after 3 months. (C) Heatmap showing correlation of CAL with resistin at baseline. (D) Heatmap showing correlation of CAL with resistin after 3 months. Khaki block = weak positive correlation, red block = positive correlation, and deep blue block = negative correlation, correlation depth of the color shows size of correlation coefficient,  ^∗∗∗^ = significant.

In the 2018 periodontitis classification, CAL was considered the mainstay of periodontitis staging. Therefore, an investigation of the relationship between adiponectin, resistin, and CAL was conducted in this trial. When Pearson correlation at baseline was assessed, it showed that there was no statistical significance in the correlations between CAL and adiponectin as follows: *r* = 0.3, *p* = 0.2 at mild periodontitis; *r* = 0.28, *p* = 0.3 at moderate periodontitis; *r* = 0.48, *p* = 0.06 at severe periodontitis; and finally *r* = 0.32, *p* = 0.2 at very severe periodontitis. After 3 months of periodontal therapy, Pearson correlation showed no statistical significance between CAL and adiponectin: *r* = 0.46, *p* = 0.08 at mild periodontitis; *r* = 0.01, *p* = 0.99 at moderate periodontitis; *r* = −0.41, *p* = 0.1 at severe periodontitis; and *r* = −0.54, *p* = 0.04 at very severe periodontitis. Concerning resistin, there was no statistical significance correlations between CAL and resistin in both baseline or 3 months after periodontal therapy as following; *r* = 0.45 *p* = 0.09 in baseline of mild group, *r* = 0.31*p* = 0.2 in baseline of moderate group, *r* = 0.24*p* = 0.3 in baseline of severe group, *r* = 0.1*p* = 0.9 in baseline of very severe group, *r* = 0.28 *p* = 0.3 after 3 months of mild group, *r* = 0.12 *p* = 0.6 after 3 months of moderate group, *r* = −0.27 *p* = 0.3 after 3 months of severe group, and lastly *r* = 0.22 *p* = 0.4 after 3 months of very severe group (Figure 2).

### 3.9. Comparison of Resistin and Adiponectin in Grade B Versus Grade C Periodontitis, Prior to and Following Periodontal Therapy

**Figure 3:**
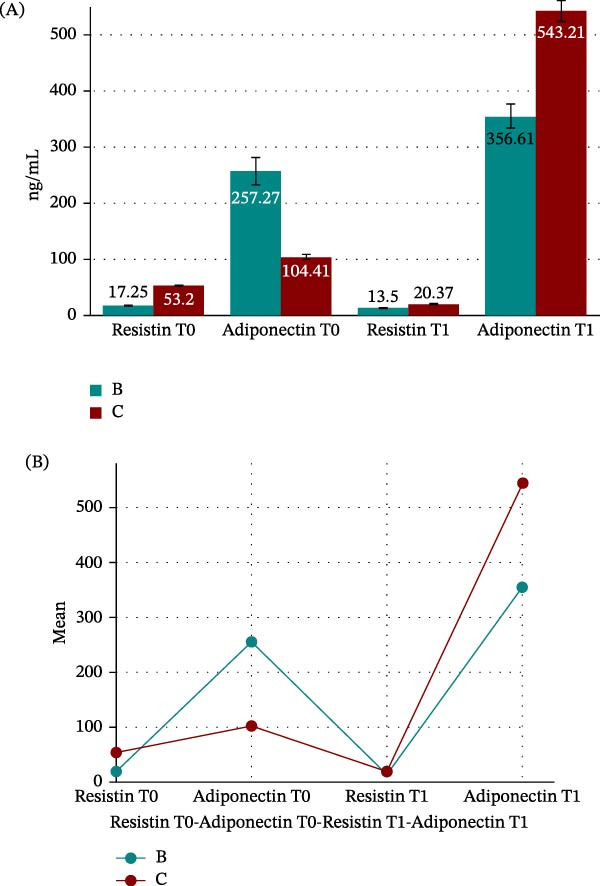
(A) Bar chart showing mean values of adiponectin and resistin in grade B versus grade C in both intervals baseline and 3 months after periodontal therapy. (B) Interaction plot showing adiponectin has distinguishing characteristics between grade B and grade C.

In grade B periodontitis patients, resistin decreased from 17.25 ± 6.1 ng/mL at study inception to 13.5 ± 1.39 ng/mL after periodontal therapy. On the contrary, adiponectin rose from 257.27 ± 84.26 to reach up to 355.61 ± 72.4 ng/mL. In the grade C periodontitis group, resistin levels ranged from 53.2 ± 1.16 ng/mL to depleted levels of 20.37 ± 2.03 ng/mL after completion of periodontal therapy. Conversely to resistin, adiponectin, with a lesser amount of 104.41 ± 8.87 ng/mL at the initiation of this study, was elevated after 3 months of periodontal control to a greater amount of 543.21 ± 36.81 ng/mL. Mixed model ANOVA showed that there was a significant difference between the groups of the independent variable grades in relation to the dependent variable *p* = 0.009, that there was a significant difference between the groups of the independent variable resistin T0‐adiponectin T0‐resistin T1‐adiponectin T1 in relation to the dependent variable *p*  < 0.001 and that there was an interaction between the two variables grades and resistin T0‐adiponectin T0‐resistin T1‐adiponectin T1 in relation to the dependent variable, *p*  < 0.001. In a more detailed post hoc test for comparing resistin and adiponectin across two intervals with both grades (B and C), there was no statistically significant difference for resistin. In contrast, adiponectin showed a high statistically significant difference between grade B and grade C at the beginning of the study and after 3 months of periodontal therapy (Table 5, Figure 3).

**Table 5:** Comparison of resistin and adiponectin between grade B versus grade C, prior and furthermore periodontal therapy.

	Grade	Variance			Interquartile range				Mean ± std.		
Resistin T0	B	37.2			11.99				17.25 ± 6.1		
	C	1.33			0.98				53.2 ± 1.16		
Resistin T1	B	1.94			1.64				13.5 ± 1.39		
	C	4.11			1.77				20.37 ± 2.03		
Adiponectin T0	B	7100.09			138.1				257.27 ± 84.26		
	C	78.7			8.96				104.41 ± 8.87		
Adiponectin T1	B	5242.18			91.02				355.61 ± 72.4		
	C	1354.62			64.17				543.21 ± 36.81		

Mixed model ANOVA for periodontitis grades vs. resistin											
		SS			df		MS		*F*		*p*
Resistin T0, resistin T1		3646.5			1		3646.52		321.04		0.00 ^∗^
Grades		10313.9			1		10313.95		524.98		0.00 ^∗^
RM factor vs. grades		4756.6			1		4756.62		418.78		0.00 ^∗^

Mixed model ANOVA for periodontitis grades vs. adiponectin											
				SS	df		MS		*F*		*p*
Adiponectin T0, adiponectin T1				1009681.2	1		1009681.28		143.53		0.00 ^∗^
Grades				6787.5	1		6787.5		2.54		0.11
RM factor vs. grades				652006.4	1		652006.43		92.69		0.00 ^∗^

Bonferroni post hoc tests—grades											
		Mean difference				SE			*t*		*p*
B versus C		−19.39				7.356			−2.64		0.00 ^∗^

Bonferroni post hoc tests—grades											
				Mean difference				Standard error		*t*	*p*
Resistin T0–B vs. resistin T0‐C				−35.95				14.712		−2.4	0.4
Resistin T0–B vs. resistin T1‐B				3.76				10.403		0.36	0.9
Resistin T0–B vs. resistin T1‐C				−3.12				14.712		−0.2	0.9
Resistin T0–C vs. resistin T1‐B				39.71				14.712		2.7	0.2
Resistin T0–C vs. resistin T1‐C				32.83				18.019		1.8	0.9
Resistin T1‐B vs. resistin T1‐C				−6.87				14.712		−0.4	0.9
Adiponectin T0‐B vs. adiponectin T0‐C				152.86				14.712		10.3	0.00 ^∗^
Adiponectin T0‐B vs. adiponectin T1‐B				−98.34				10.403		−9.45	0.00 ^∗^
Adiponectin T0‐B vs. adiponectin T1‐C				−285.94				14.712		−19.4	0.00 ^∗^
Adiponectin T0‐C vs. adiponectin T1‐B				−251.2				14.712		−17.1	0.00 ^∗^
Adiponectin T0‐C vs. adiponectin T1‐C				−438.8				18.019		−24.3	0.00 ^∗^

*Note:* T0—baseline, T1—after 3 months, A—group І, B—group Ⅱ, C—group Ⅲ, D—group Ⅳ.

Abbreviations: ANOVA, analysis of variances; df, degrees of freedom; MS, mean square; SS, sum of squares.

^∗^ = ≤0.05 Significant.

## 4. Discussion

Periodontitis is a prevailing erythrogenic riposte to interlacing microbial plaque with host inflammatory arbitrators, ensuing periodontitis. Due to an excessive accumulation of body fat on obese, adipose tissue elaborate biologically effective adipocytokines, which are associated with persistent adiponectin and resist in levels in obese individuals. A little while back, Adipokines were discovered as they were expressed at a very low level in adipocytes and at a higher level in circulating inflammatory immune cells. Periodontitis coveted burden obese patients ubiquitously than non‐obese persons [15]. So, the present scrutiny was designed as single blinded clinical trial to appraise the relationship between periodontitis and GCF adiponectin and resist in obese patients as well as figure out the consequences of phase І periodontal therapy on their levels. Patients were selected in this study are obese, as mentioned in Nascimento et al. [16] review shown that obese individuals have a 35% higher risk of developing periodontitis compared to normal‐weight individuals. Class 3 obesity patients were participated in this trial according to Malik et al. [17] review which reporting that class 3 obese patients suffering from periodontitis with high severity and affected a higher number of teeth, or with a high periodontitis prevalence of up to 67% and 70.8%. In this trial, adiponectin and resistin were assessed as they involved in both diseases’ obesity and periodontitis. This statement is consistent with previous studies of both Boyapati et al. [18] and Suvan et al. [19] they mentioned that obesity‐related disorders are tightly linked to low‐grade chronic inflammation e.g. periodontitis as adiponectin has defensive effects due to suppression of tumour necrosis factor‐α and interleukin‐6, along with induction of interleukin‐1 receptor antagonist also resistin are mainly involved in obesity, diabetes mellitus and involved in chronic periodontitis by acting as a proinflammatory molecule stimulates the synthesis and secretion of TNF‐α and IL‐12. Pregnant and medically compromised patients were excluded, because it was reported by Gudnadóttir et al. [20] that accuracy of results of clinical trials may be affected by these diseases and conditions. Hiroshima et al. [21] estimated the amount of resistin in GCF, serum and synovial substance and noticed resist in collection in GCF was exceedingly higher‐up than serum and synovial fluid. So, GCF sampling had been utilized in the current trial. This trial showed that elderly obese patients more correlated with periodontitis, these investigations concurrent with systemic review of Kim et al. [22] they mentioned that elderly individuals are more vulnerable to periodontitis than middleaged. Consequently, they are vulnerable to the sequela of obesity. Type ІІІ obese patients participated in contemporary study and that’s because Obese individuals have a higher risk of developing severe periodontitis, but individuals with periodontitis also tend to have a higher BMI as reported in Lê et al. [23] study. There was agreement of this trial results of positive correlation of BMI and HbA1c with observations of Babikr et al. [24] study that illustrated that there is a statistically significant positive correlation between HbA1c and BMI (*r* = 0.240, *p* < 0.0001). In this present study, the analysis showed significant positive correlation between MBI and irreversible periodontal indicators (CAL, PD). This notices in accordance with Çetin et al. [25] study which discovered a statistical relationship between BMI and clinical attachment loss, probing pocket depth, plaque index, stage and grade of periodontitis, and the number of remaining teeth. It was concluded that BMI increases the risk of developing stages III and IV of periodontitis. In contrary to this trial results of relationship between BMI & clinical parameters after periodontal therapy, Suvan et al. [26] trial concluded that high BMI status was independently predictive of a worse response to periodontal therapy as assessed by periodontal status at 2 months. Concerning to levels of adiponectin and resistin, Fairlin et al. [27] investigations showed that A statistically significant decreasing of resistin was observed after periodontal therapy while adiponectin showed statistically significant increasing after periodontal therapy. Their findings in harmonious with current study. There was consonant with study of Furugen et al. [28] found that serum resistin levels (positive correlation), but not serum adiponectin levels (negative correlation), were associated with periodontal inflammation severity. The sitting study estimated the correlation of CAL with adiponectin and resistin as CAL considered as cornerstone of periodontitis staging. In other side, Boraha et al. [29] study didn’t investigate these correlations. Mahmood and Zardawi [30] mentioned that correlation between the salivary resistin and PPD and CAL was a negative weak, not significant correlation, this is consistent with current results. Also, clinical trial of Devanoorkar et al. [31] mentioned not much difference in serum resistin levels between healthy and periodontitis persons and decreased resistin levels following nonsurgical periodontal therapy did not show any statistically significant difference. in contrary to current study, Varghese et al. [32] study concluded that obese subjects showed significant correlation of clinical periodontal parameters with resistin after non‐surgical periodontal therapy. Opposite to results of present trial about adiponectin standards were reported by Duzagac et al. [33] study that summarized non‐surgical periodontal therapy not significant, improved GCF adiponectin levels after 3 months in periodontitis individuals with obesity. They mentioned that non‐significant, the increased adiponectin levels observed in, even though obesity is usually associated with hypoadiponectinemia, is a valuable finding and shows the important role of the management of obese patients with periodontitis. In contrast to this research findings, Gonçalves et al. [34] reported decreased serum adiponectin levels at 3 months after therapy in obsess patients with periodontitis. Study of Abdellatif et al. [35] illustrated that no correlation existed between salivary adiponectin and clinical periodontal parameters (PI, GI, PD and CAL) before or after therapy, this is in accordance with the investigations of this present study. While this trial based on 2018 classification on periodontitis grading, the most recent systemic review in benefits of periodontal therapy in obese patients with periodontitis with huge database showed no trials manoeuvred system of grades of periodontitis. So far it seems, the staging of periodontitis has not manipulated in extensive research [36].

### 4.1. Challenges and Future Research Prospects

One limitation of this current trial was the absence of a control group, such as healthy obese individuals or normal‐weight patients with periodontitis, which restricts the ability to isolate the independent effects of obesity and periodontal disease on adipokine expression. Another limitation is the relatively small sample size within each subgroup (*n* = 15), which may reduce the statistical power and limit the generalizability of the findings. In addition, the study assessed only two adipokines (adiponectin and resistin), which does not fully capture the complexity of the inflammatory and metabolic networks involved in obesity‐associated periodontitis.

## 5. Conclusions

Adiponectin and resistin, as anti‐inflammatory and proinflammatory mediators, respectively, were significantly affected after periodontal therapy, especially in stages 3 and stage 4 periodontitis. Significant improvements in clinical periodontal parameters were observed following phase I periodontal therapy across all study groups. A clear association was also identified between increasing BMI and HbA1c levels and the severity of periodontitis. Also, adiponectin demonstrated a statistically significant difference between grade B and grade C periodontitis.

**Nomenclature**
GCF: Gingival crevicular fluid
BMI: Body mass index
HbA1c: Glycated hemoglobin
RELMs: Resistin and its family resistin‐like signaling molecules
CRP: C‐reactive protein
ELISA: Enzyme‐linked immunosorbent assay
OD: Optical density
PIs: Plaque scores
BoP: Bleeding on probing
PPD: Probing pocket depth
CAL: Clinical attachment loss
NSAID: Antimicrobial/nonsteroidal anti‐inflammatory
N: Newton
Ng: Nanograms
PBS: Phosphate‐buffered saline.

## Author Contributions

Conceptualization: Bahaa Mohammed Badr, Asem Mohammed Kamel, Shuaibu Abdullahi Hudu, and Asmaa Rashad Ali. Formal Analysis: Asem Mohammed Kamel, Bahaa Mohammed Badr, Ibrahim Hammad Ibrahim, and Abdullah Ibrahim Ali. Investigation: Ibrahim Hammad Ibrahim and Abdullah Ibrahim Ali. Methodology: Ibrahim Hammad Ibrahim, Abdullah Ibrahim Ali, and Shuaibu Abdullahi Hudu. Experimental studies: Ibrahim Hammad Ibrahim and Abdullah Ibrahim Ali. Project administration: Asem Mohammed Kamel, Bahaa Mohammed Badr, Ibrahim Hammad Ibrahim, and Shuaibu Abdullahi Hudu. Writing – original draft: Asem Mohammed Kamel, Bahaa Mohammed Badr, Asmaa Rashad Ali, and Shuaibu Abdullahi Hudu. Writing – review and editing: Bahaa Mohammed Badr, Asem Mohammed Kamel, Asmaa Rashad Ali, and Shuaibu Abdullahi Hudu.

## Funding

No funding was received for this manuscript.

## Ethics Statement

The Helsinki Declaration ethical guidelines were followed in this trial, as its registration was completed in the ClinicalTrials.gov Protocol Registration and Results System under ID NCT06604013. It was approved by the ethics committee of the Faculty of Dentistry, Al‐Azhar University, Assiut (Approval Number: AUAREC20230009‐4).

## Consent

All contributors were adults and provided written consent before the collection of the experimental samples.

## Conflicts of Interest

The authors declare no conflicts of interest.

## Data Availability

Data will be made available upon request from the authors.
